# Oral Appliance Therapy for Obstructive Sleep Apnoea: State of the Art

**DOI:** 10.3390/jcm8122121

**Published:** 2019-12-02

**Authors:** Kate Sutherland, Peter A. Cistulli

**Affiliations:** 1 Sleep Research Group, Charles Perkins Centre and Northern Clinical School, Faculty of Medicine and Health, University of Sydney, Sydney, NSW 2006, Australia; peter.cistulli@sydney.edu.au; 2 Department of Respiratory Medicine, Royal North Shore Hospital, Sydney, NSW 2065, Australia

**Keywords:** obstructive sleep apnoea, oral appliance, treatment effectiveness

## Abstract

Obstructive sleep apnoea (OSA) represents a significant global health burden, with impact on cardiometabolic health, chronic disease, productivity loss and accident risk. Oral appliances (OA) are an effective therapy for OSA and work by enlarging and stabilising the pharyngeal airway to prevent breathing obstructions during sleep. Although recommended in clinical guidelines for OSA therapy, they are often considered only as second-line therapy following positive airway pressure (PAP) therapy failure. There has been a long-standing barrier to selecting OA over PAP therapy due to the inability to be certain about the level of efficacy in individual OSA patients. A range of methods to select OSA patients for OA therapy, based on the outcome of a single sleep study night, have been proposed, although none has been widely validated for clinical use. Emergent health outcome data suggest that equivalent apnoea–hypopnea index reduction may not be necessary to produce the same health benefits of PAP. This may be related to the more favourable adherence to OA therapy, which can now be objectively verified. Data on longer term health outcomes are needed, and there are additional opportunities for device improvement and combination therapy approaches. OAs have an important role in precision care of OSA as a chronic disorder through a multi-disciplinary care team. Future studies on real-world health outcomes following OA therapy are needed.

## 1. Introduction

Obstructive sleep apnoea (OSA) is characterised by the repetitive collapse of the pharyngeal airway during sleep leading to complete or partial airflow obstruction, resulting in perturbations (i.e., intermittent hypoxia, sleep fragmentation, intra-thoracic pressure swings) which provide mechanistic links to a range of neurobehavioral and cardio-metabolic consequences. OSA is an independent risk factor for a number of conditions including hypertension, cardiac disease, stroke, type 2 diabetes, as well as motor vehicle accident risk and all-cause mortality [1,2,3,4,5]. OSA is common, and recent estimates suggest over one billion people globally are affected [6].

Although a potential global health crisis, OSA management is challenging. Positive airway pressure (PAP) therapy has long been the mainstay of treatment and is still first line in the majority of cases, particularly as OSA severity increases. PAP is highly efficacious in preventing pharyngeal collapse while in use; however, improvement in health outcomes is dependent on the number of hours of nightly usage [7,8]. Although many OSA patients successfully use PAP in the long term, many others are suboptimal PAP users, limiting real-world effectiveness and creating a need for alternate treatment options. The most common alternative OSA treatment device is an oral appliance (OA). Evidence-based clinical guidelines recommend OA for OSA if PAP therapy is not successful or if a patient prefers OA treatment, regardless of OSA severity [9]. Our understanding of the effectiveness of OA as an OSA therapy has increased over many years of research. This review aims to provide an up-to-date overview of OA therapy for OSA.

## 2. Mechanism of Action

OAs can be broadly classified as devices that protrude the tongue (tongue-retaining devices, TRD) and devices that advance the lower jaw (commonly termed mandibular advancement splints or devices, MAS or MAD). Although there is evidence for TRD use in the treatment of OSA [10], and this may be the only option in some edentulous patients, clinical guidelines specifically relate to mandibular advancement devices [9]. It is these devices which are discussed in this review.

**Figure 1:**
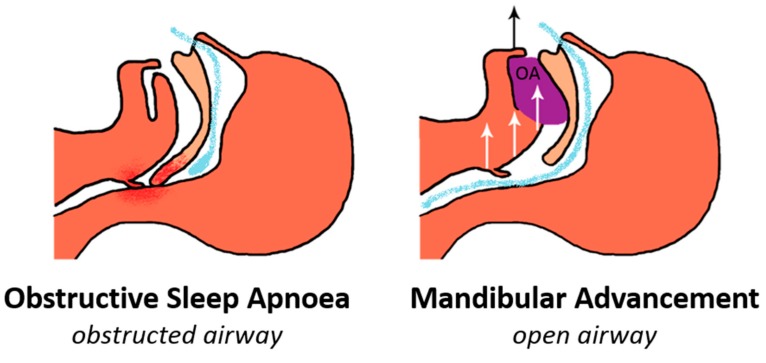
Oral appliance (OA) therapy for obstructive sleep apnoea (OSA). OAs allow the mandible to be retained in a forward position relative to the maxilla. This action enlarges and stabilises the pharyngeal airway, preventing pharyngeal collapse and obstructed breathing.

Protrusion of the lower jaw is the essential therapeutic mechanism of action [11] (Figure 1), which has been shown to enlarge the airway space, as visualised by MRI and nasopharyngoscopy [12,13]. The most prominent feature is a widening of the lateral airway diameter, particularly in the velopharyngeal region (behind the soft palate) [12,13], likely through soft tissue connections between the lateral airway wall muscles and the mandible [14]. The tongue shows evidence of anterior repositioning with the mandibular advancement, although to varying degrees in different individuals [14]. In addition, the mandibular advancement decreases the upper airway collapsibility, variously demonstrated using pharyngeal critical pressure (P_crit_) [15,16] and pharyngeal closing pressure (P_close_) methods [17], optimal positive airway pressure (PAP) pressure (a surrogate measure of P_crit_) [18] and reduction in spontaneous collapse during sedation [19]. This stabilisation of pharyngeal airway is likely largely attributable to the anatomical enlargement of the airway and the anterior movement of the tongue [13,14].

Whether there are additional direct effects on pharyngeal dilator muscle activity is less clear. The activity of the major pharyngeal dilator, the genioglossus, in response to mandibular advancement has only been assessed in limited studies. Using intra-oral surface or intra-muscular fine-needle electrodes, an increase in genioglossus activity with mandibular advancement has been observed during wakefulness [20,21]. An increase in muscle activity could contribute to the effectiveness of the device. In a single case, intra-oral surface electrodes showed a decrease in both tonic and phasic genioglossus activity with progressive mandibular advancement during sleep [22], suggesting the muscles could switch off in response to the more favourable anatomy created by the advancement. In a slightly larger study of 12 participants during sleep, intramuscular fine-needle electrodes detected no overall effect of progressive mandibular advancement on genioglossus muscle responsiveness to induced apnoea or no effectiveness in restoring flow [15]. This would support a purely passive anatomical mechanism of action. However, this experimental condition required participants to simultaneously be on therapeutic PAP to maintain airflow, and the muscles could therefore be in a passive state. It is yet unclear if this reflects the behaviour of the genioglossus response to mandibular advancement in the natural sleep state. However, it is evident that OA reduces pharyngeal collapsibility and improves airway geometry.

## 3. Efficacy of Oral Appliance Therapy

OA improves indices of OSA as measured by polysomnography. Respiratory event indices as well as oxygen saturation, arousal index and sleep efficiency have demonstrated improvements compared to their baseline values in a sleep study [23]. The clinical metric to categorise OSA presence and severity is the apnoea–hypopnea index (AHI). It has long been recognised that the average AHI reduction induced by OA therapy use is less than that seen when PAP is applied, with residual OSA often present (AHI > 5 events/hour) [24]. Unlike PAP, which will lead to AHI normalisation if applied at adequate pressure, OA use is results in a large inter-individual variability of the AHI [23]. An example of efficacy variation from a sample of 425 OSA patients treated with a customized OA is shown in Table 1. The definition of an OA “responder” varies in the literature, from a complete response (AHI < 5, or no OSA) to a minimum 50% AHI reduction from baseline (often termed a ‘partial response’) and definitions related to values between these two extremes (e.g., AHI <10 plus 50% reduction from baseline). These three definitions are most commonly presented, but additional definitions also exist. On average, AHI reduction across the range of OSA severity is around 50%, but with a wide variation [23]. Approximately two-thirds of OA-treated patients will achieve greater than 50% reduction in AHI, with at least one-third achieving a complete response [23]. Therefore, many OSA patients will receive a highly efficacious OSA therapy with an OA alone. However, uncertainty around the amount of AHI reduction an individual will achieve has been a barrier to the implementation of this mode of therapy, and OA is still commonly reserved for second-line therapy, particularly in more severe OSA.

## 4. Factors Related to Oral Appliance Efficacy and Prediction Methods

The uncertainty around the level of AHI reduction achieved following OA therapy has remained a clinical barrier to the implementation of OAs in routine clinical practice. A clinical prediction method has been a focus of much research, but this ‘holy grail’ remains elusive. A complexity in comparing prediction studies is the variation in treatment response definitions. Many studies have started to present the three commonly used definitions of treatment response (described above) to improve generalisability, but others use a single definition. Understanding individual variations in efficacy has been the focus of much research [25]. A summary of prediction methods and their performance is given in Table 2. Group clinical characteristics such as less obesity, lower age, and female gender are associated with treatment success [23,26,27]. A higher baseline AHI is a feature of non-responders at the group level, but nearly a quarter of severe OSA patients will achieve a complete response (AHI < 5) [23]. While clinical and polysomnographic information reveals characteristic patterns for treatment responders and non-responders, these are not adequate predictors to rule someone in or out for therapy [23,28,29]. Therefore, there is a need for additional tests or information above routine clinical information.

Craniofacial structure, predominately assessed by lateral cephalometry, is associated with OA treatment response. However, there is very little consistency between studies of craniofacial features, which vary in sample populations, treatment response definitions and craniofacial measurements assessed [29,30]. Cephalometric investigations include skeletal and dental measurements, upper airway soft tissues and airway space. Even features most commonly reported as beneficial, such as hyoid bone position, cranial base angle, soft palate length, retruded mandible, have no association in other studies [29,30]. There is some suggestion that the amount of soft tissue relative to craniofacial bony enclosure (‘anatomical balance’ [44]) relates to treatment response [45,46], but this requires further exploration. Oropharyngeal crowding, assessed by the Mallampati score, particularly when combined with obesity, has been identified as a negative predictor [31]. A posteriorly located tongue observed during natural sleep by nasendoscopy has also been noted as an anatomical marker of favourable response [16].

Some physiological features have also been associated with greater OA efficacy. A more collapsible pharyngeal airway at baseline is a negative indicator [42]. Optimal PAP pressure, a surrogate for pharyngeal collapsibility [47], has predictive value, with higher pressure requirement associated with greater likelihood of being a non-responder [33,34,35,48]. However, the cut-off pressure values for identifying non-responders differ somewhat between studies, as does its predictive value, with or without other clinical variables. This could reflect differences in pressure titration methods, with ethnic and gender differences in the studied populations likely being an important factor.

The site of pharyngeal collapse, oropharyngeal rather than velopharyngeal collapse, is predictive of a good treatment response [39]. Different methods for estimating the site of collapse have been assessed as prediction methods. Nasendoscopy to observe pharyngeal behaviour during induced sleep (drug-induced sleep endoscopy, DISE) has shown collapse behind the palate to be a positive indicator of OA treatment response, while hypopharyneal collapse tends to be a negative indicator [19]. An awake assessment of the site of collapse by measuring velopharyngeal and oropharyngeal pressure via a pharyngeal catheter (under condition of phrenic nerve stimulation to simulate pharyngeal obstruction during sleep) found oropharyngeal collapse to be associated with complete response [49]. Flow-volume loop metrics were explored as a surrogate measure of site of airway collapse and showed predictive utility for OA outcome in a derivation sample [36]. However, this was not able to be validated in a subsequent sample [37], illustrating the importance of the validation of prediction models derived from small clinical samples.

The technique of observing changes in the pharyngeal airway with mandibular advancement via nasendoscopy has also been applied during wakefulness [12], a simpler assessment than DISE in some clinical environments. A qualitative assessment of pharyngeal changes with mandibular advancement and performance of the Mueller manoeuvre on a scoring chart found inadequate discrimination between responders and non-responders for prediction [50]. A Japanese prediction study used voluntary mandibular protrusion to measure the pharyngeal cross-sectional area (CSA) at different levels using an image analysis software [38]. The CSA during mandibular advancement was compared to the CSA with centric occlusion (CSA expansion ratio). This ratio in the velopharyngeal region, in combination with baseline AHI, had predictive utility across the three response definitions, with accuracy varying across prediction models (area under the receiver operating characteristic (ROC) curve ranging from 0.74 to 0.87).

In recent years, there has been interest in non-anatomical contributors to pharyngeal obstruction [51]. One of these traits, the stability of the ventilatory system, is quantified using a value termed ‘loop gain’. A lower loop gain, or more stable ventilatory control, has been associated with treatment response in long-term user of the treatment, alone and in combination with less collapsibility [42]. Recent work has estimated pathophysiological traits, or endotypes, both pharyngeal (collapsibility and muscle compensation) and non-pharyngeal (loop gain, arousal threshold, ventilator response to arousal) traits, using algorithms applied to clinical polysomnography. This study found a complement of traits that showed some relationship to OA treatment response, including lower loop gain, higher arousal threshold, moderate pharyngeal collapsibility and weaker muscle compensation. However, its predictive utility was not above that of other published models, and there remains a need for prospective validation before any clinical utility can be confirmed.

Most prediction models add data from a single type of assessment to clinical information, which may not capture the complexity of OA action in preventing sleep-disordered breathing. A multimodal phenotyping approach was assessed as a way to improve prediction by combing assessments of site of collapse surrogates (flow-volume loop), craniofacial structure, and nasendoscopy investigation [52]. The prediction modelling was structured using clinical information (age, obesity, AHI) as readily available variables and subsequently assessed the benefit of adding the results from three phenotypic tests. Although multimodal phenotyping improved the prediction model, the magnitude of the improvement may not justify the additional effort and cost. These models were not tested beyond the original derivation sample, but accuracy ranged from fair to excellent, depending on which response definition was used.

A question often asked is whether an inexpensive, ‘off-the-shelf’ or non-customised appliance can be used to indicate treatment outcome with a customised device. No studies have been specifically designed to assess a prefabricated non-customised appliance as a prediction tool. However, two studies have compared a customised and a non-customised appliance in a randomised cross-over design [53,54]. In one study, 63% of patients who did not receive any benefit using the non-customised device were treatment responders when using the customised device [53]; in another study, 44% of patients who did not respond to a non-customised device were treatment responders to customised ones [54]. This suggests that the results from a night study using a non-customised device are not comparable to those of a study with a customised device. Another a sleep test method is based on the use of a remotely controlled mandibular protrusion device enabling the manipulation of the jaw position to eliminate obstructive events in real time during sleep monitoring, additionally determining the amount of advancement required [55,56,57]. There have been two studies using prototype devices [55,56], and two studies using a commercial remotely control mandibular protrusion (RCMP) device [28,40]. The commercial device used in-laboratory polysomnography and a prediction algorithm based on events in supine REM (rapid eye movement) sleep, which is essential to its predictive capability [28,40]. Predictive accuracy was shown to be relatively stable by two laboratories, but higher test failure rates and less capacity to identify a sub-maximal mandibular protrusion level were found in a validation study by an independent sleep laboratory [40]. A home-testing approach has also been assessed, using a feedback-controlled mandibular positioner for two nights [58].

To date, there are no robust and widely validated prediction methods [25]. Other issues to keep in mind in regard to the achievement of a universal prediction model for OA therapy, is that the outcome is based on the total AHI on a single night. We know AHI varies in polysomnography scoring, from night to night and depending on sleep stage and body position [59,60]. Additionally, its efficacy varies over time (e.g., with weight gain) [61,62]. Therefore, prediction of efficacy, based on an initial, single, sleep study night, is not a robustly accomplished, and this has likely contributed to the limited success of the prediction models. An additional complication is that we do not know the level of efficacy that is needed to attain the desired health outcomes, and treatment adherence has an important influence on treatment effectiveness.

## 5. Treatment Effectiveness of Oral Appliance Therapy

It is increasingly recognised that AHI changes have limitations in terms of indicating the health benefits of OSA therapy [63]. Ultimately, the goal of OSA therapy is to improve symptoms, and sleep quality and reduce health risks. Efficacy (assessed by the AHI on a single treatment night) reflects the outcome of a treatment administered under ideal circumstances and is only one component of treatment effectiveness. How OSA treatment is administered in the real world has impact on health outcomes. In the case of PAP, hours of nightly usage have been shown to be important in evoking positive health changes in terms of sleepiness, daily functioning, blood pressure and cardiovascular disease [7,8,64].

There is growing evidence that despite residual AHI with OA therapy versus PAP, inferior health outcomes do not necessarily result. For example, CPAP has a well-documented small but significant effect in reducing blood pressure (in the order of 2 mm Hg, greater in resistant hypertension) [65,66]. A recent network meta-analysis has confirmed that both PAP and OA therapies reduce short-term blood pressure to the same extent versus respective sham therapies, with no difference in blood pressure reduction between therapies [7]. A short-term randomised controlled trial of one month of optimised OA and PAP therapies assessed a range of health outcomes (cardiovascular symptoms, quality of life, driving simulator performance) after each therapy period in a sleep clinic population enriched in severe OSA cases [67]. OA therapy was found to be non-inferior to PAP across all outcomes and even superior to it for some domains of health-related quality of life. As predicted, AHI with PAP treatment was reduced to normal levels, while mild residual OSA was evident with OA therapy [67]. However, OA therapy was reported to be used longer than PAP therapy during the trial (by >1 h night), and more participants chose to continue using OA therapy at the end of the trial.

**Figure 2:**
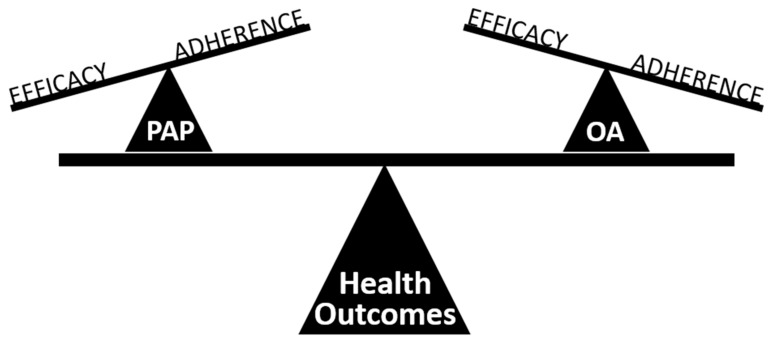
Treatment effectiveness and treatment profiles of OA, and standard PAP therapy. There is evidence that at least the short-term health outcomes of OA and PAP are similar, despite mild residual sleep apnoea with OA treatment. Although PAP is highly efficacious, adherence to it outside of the sleep laboratory is often suboptimal. Treatment effectiveness, in terms of health benefits, is a composite of efficacy and adherence. OA and PAP have different profiles of efficacy and adherence. However, the end result in terms of treatment effectiveness may be the same.

The potential explanation for equivalent health outcomes is that a highly efficacious therapy with moderate usage (PAP) results in the same effectiveness as a moderately efficacious therapy with higher usage (OA) [68] (Figure 2). Therefore, adherence is a key component of treatment effectiveness, which is not captured by traditional metrics of treatment efficacy (AHI). Metrics which incorporate time on and off treatment to describe treatment effectiveness have been proposed and evaluate ‘mean disease alleviation’ or ‘sleep-adjusted residual AHI’ [68,69,70,71]. However, formulas which aim to adjust for adherence over longer treatments are reliant on some assumptions, particularly, that AHI reverts to diagnostic polysomnography levels when therapy is removed. This is not true at least for PAP, for which it has been demonstrated that AHI does increase in partial users when PAP treatment is removed in the night although AHI may not revert to baseline levels, AHI values can still correspond to severe OSA [72]. An in-laboratory study of the objective measurement of AHI in partial PAP users has shown that effectiveness measures could be collected using simultaneous measurements with a portable monitor [72]. New wearable technologies for monitoring of sleep-disordered breathing, sleep time, and treatment compliance, may in the future allow objective treatment effectiveness measures to be explored in relation to health outcomes.

## 6. Cardiovascular Health

OSA is an independent risk factor for hypertension and cardio- and cerebro-vascular diseases including coronary heart disease, heart failure and stroke [1,73,74,75]. However, demonstrating a beneficial effect of OSA treatment on cardiovascular endpoints has proved elusive. The largest randomised study of PAP therapy (the Sleep Apnoea Cardiovascular Endpoints, or SAVE, study), with an average of 3.7 years follow-up, showed no benefit of PAP in secondary prevention of composite cardiovascular events [76]. One of the potential confounders to this trial was that average PAP usage was only 3.3 h/night in this cardiovascular disease population. A recent meta-analysis of patients using PAP for more than 4 h per night, found a positive effect on the occurrence of a composite outcome including stroke, cerebrovascular disease, cardiac death or acute myocardial infarction [64]. This suggests OSA treatment adherence is key in cardiovascular prevention. It is unknown whether OA therapy may be a more acceptable therapy in cardiovascular clinical populations and therefore may have a potential role in cardiovascular disease prevention.

A systematic review [77] has summarised the cardiovascular effects of OA therapy. As discussed above, blood pressure reduction appears to be similar to that achieved with PAP [7]. A single observational study suggests OA therapy is as effective as PAP in reducing the risk of death related to cardiovascular mortality [78]. In terms of intermediary markers of cardiovascular health (biomarkers, heart rate variability, arterial stiffness, endothelial function), most studies are very small and short-term and have heterogeneous designs, which makes any results about OA effects inconclusive [77]. A more recent randomised controlled trial of 6 months OA treatment vs sham treatment (single plate providing no mandibular advancement) did not find a beneficial effect on microvascular endothelial function assessed by the reactive hyperaemia index [79]. However, the study population was relatively healthy from a vascular point of view at baseline and free of cardiovascular disease. Therefore, studies of populations with cardiovascular risk factors are warranted. More trials of intermediary cardiovascular markers are needed to assess the potential of OA therapy in this field.

## 7. Devices and Customisation

With more than 150 devices with FDA approval for the treatment of snoring and OSA patients, there is considerable debate about the efficacy of different devices. In reality, only a small number of commercially available devices have been subjected to robust clinical scrutiny, and head-to-head comparison studies are also limited. Customised devices which are titratable (allow adjustment of the mandibular advancement) and implemented by a qualified dentist are suggested in the clinical guidelines [9]. Non-customised devices (prefabricated, ‘off-the-shelf’ devices which mould the teeth when heated) tend to be poorly tolerated and less effective than customised devices [53,54,80,81].

There is a relationship between ‘dose’ of mandibular advancement and effects of sleep-disordered breathing, with further advancement generally leading to better results. However, there is also some evidence that greater advancement may not always provide further benefit. There may be some individuals that have most benefit at a submaximal level of mandibular advancement, for whom further advancement is detrimental [15,18,82]. Avoiding unnecessary advancement may be a way to reduce side effects such as tooth movements and improve treatment acceptance.

The methods used for OA titration vary and include mandibular advancement to patients’ physical limits, monitoring symptom resolution, or using polysomnography or single-channel devices [83,84]. Other proposed titration protocols have used nasendoscopy to observe effects on the pharyngeal area for different levels of mandibular advancement (provided by an occlusal registration tool) [85]. The RCMP device has been used during both polysomnography and DISE to work out the effective therapeutic target protrusion, and was successful for most, but not all, patients in these studies [86].

Another parameter distinguishing different OAs regards the vertical dimension, i.e., the amount of mouth opening permitted by the device. Vertical opening without mandibular advancement has been shown to have a negative effect on AHI [87] and pharyngeal dimensions in some patients [88]. In the context of mandibular advancement, a cross-over study examining the effect of 4 mm vs. 14 mm of vertical mouth opening showed no effect of vertical height on AHI improvement [89]. However, patients preferred the reduced opening, suggesting that vertical dimension may impact patient acceptance and adherence. Permissible mouth movement is another design difference for OAs. Some two-piece appliances allow the lower jaw to move, while one-piece (monobloc) appliances or some two-pieces with an anterior titration apparatus, for example, do not permit vertical jaw movement. There is some suggestion that devices which allow mouth opening may be detrimental for OSA patients in the supine position. Controlling the amount of permissible mouth opening, such as with elastic bands, may be important in some patients, particularly those with supine-predominant OSA [90,91].

In recent years, devices are emerging which rely on computer-aided design or computer-aided manufacturing technology. Instead of the traditional impressions, digital intraoral scans can now be used to design appliances, and digital manufacturing techniques are increasingly being used. These can streamline the process and result in more rapid access to treatment and better fit of appliances, which could improve acceptance and efficacy [92].

## 8. Long-term Side Effects

There are initial side effects when starting an OA therapy, such as dry mouth, excessive salivation or soft tissue irritation, but these are generally temporary and resolve [93]. The forces on the teeth resulting from the action of the OA in keeping the lower jaw forward can result in tooth movements and bite changes. Some bite changes are actually favourable and, in any case, often not noticed by the patient and considered subclinical [94]. There have now been studies that track the effects of oral appliance wear on bite changes in twenty years of use [95,96,97]. These studies show that tooth movements are continuous and relate to the duration and frequency of OA use and to the mandibular advancement level [95,96]. The extent of tooth movements has some relationship to the initial bite or craniofacial structures [95,98]. Material and design may also influence the side effects. A study comparing OA devices constructed of flexible material without incisor coverage to rigid appliances with full occlusal coverage, found greater short-term tooth movement resulting in irregularity in the lower teeth in patients using the former [99]. Guidelines recommend that patients have regular follow-ups with qualified dentists to survey dental side effects or occlusal changes [9].

## 9. Objective Compliance

The observed similarity in health outcomes between OA and PAP therapies, despite the greater PAP efficacy, could be explained by a greater adherence to OA treatment. However, this has largely been based on self-reported adherence data for OA. Objective compliance monitors have become available for OA, but the literature about this is limited to date. Compliance monitors are small temperature-sensing data chips which can be embedded in the OA material. Three commercial brands have been independently tested and found to be reliable for determining usage time [100]. In a three-month study with covert objective compliance monitoring, subjective (daily diary) and objective compliance (temperature microsensor) did not differ [69]. The average nightly usage across the initial three months of OA therapy was 6.7 ± 1.3 h/day, with usage on >90% of days. At one-year follow-up, the mean usage was 6.4 ± 1.7 h/night, with 83% of patients using OA for more than 4 h on more than 70% of days, with a 9.8% discontinuation rate [101]. A randomised trial of two months of OA therapy versus sham therapy in severe OSA patients found OA use for 6.6 ± 1.4 h/night, with 96.1% compliant users, compared to sham device use for 5.6 ± 2.3 h/night, with 73.6% compliant users [79]. In this study, OA therapy was used for around 90% of the reported sleep duration, but the lack of objective sleep measurements is a limitation in assessing OA therapy usage. Although initial reports of objective compliance indicated good average usage, there are likely to be different subtypes of users, which requires further investigation and longer-term data.

Objective compliance data in the short term has some relationship to self-report snoring improvement and the presence and severity of side effects [102,103]. Interestingly, AHI improvement has not been shown to be related to short-term compliance in these small studies, neither have anthropometric data or sleepiness [103].

## 10. Combination Therapy Strategies

With increasing recognition of OSA as a heterogenous disorder and the possibility of tailoring treatments to the individuals on the basis of their pathophysiology and preferences, the field has opened up to combination therapy possibilities to address efficacy and adherence deficiencies and optimise outcomes [104]. OA and PAP have been proposed to be combined in different ways. Using both treatments simultaneously can reduce the PAP pressure requirement [105,106,107] and have an additive effect on AHI in those who are PAP-intolerant [107]. Alternatively, the treatments can be used interchangeably, according to patient preference and circumstance (e.g., travel) [108]. The combination of OA and positional therapy resulted more beneficial than either therapy alone for supine OSA patients [109]. A study of uvulopalatopharyngoplasty (UPPP) surgery for OSA treatment with or without OA therapy found greater long-term control of OSA with the combination approach compared to surgery alone [110]. Other more contemporary surgical approaches for OSA have not been assessed. However, since the two approaches of upper airway surgery and OA therapy are often associated with residual AHI, a combination therapy could be beneficial. Weight loss improves OSA and, when combined with PAP therapy, leads to greater cardiometabolic benefits than weight loss or PAP alone [111]. Obesity and weight gain are known to decrease OA efficacy [23,61], thus, combined weight loss and OA therapy could be beneficial. Recently, the combination of OA therapy and expiratory positive airway pressure (EPAP) valves has been shown to reduce AHI versus either treatment alone, albeit at the cost of decreasing sleep efficiency [112]. More studies on combination therapy approaches are needed.

## 11. Models of Care

With increasing recognition that patients with OSA are phenotypically diverse and have individual needs, preferences and values, the field is undergoing a shift in OSA management, from a focus on diagnosis to a focus on chronic care management. This mandates that treatment decisions are informed by individual patients’ values and preferences in order to optimize patients’ outcomes [113]. Since sleep medicine is highly multidisciplinary, there is a need to develop efficient and cost-effective care models, centred around the patient. In the case of OA therapy, an effective working relationship between the sleep physician and the dentist is required, founded on an understanding of their respective roles in the care process. Sleep physicians currently have the primary responsibility for diagnosis and discussion of treatment options relevant to each individual patient as well as for assessment of treatment outcomes. Dentists have the responsibility for delivering the OA therapy, including assessment of dental suitability, impressions, appliance selection and fitting, titration and side effects monitoring. Ideally, the dentists should be credentialed in dental sleep medicine. Furthermore, since dentists routinely see patients for dental health check-ups, they may play a useful role in raising patient awareness of the condition and screening modalities. As practice in the field evolves, we can expect to see interdisciplinary models of care in which physicians and dentists may work within the same clinic, alongside other disciplinary experts (e.g., surgeons, psychologists). With adequate training and credentialing, it is possible that the scope of practice for dentists and other practitioners will expand in the future to help address the enormous burden of OSA.

## 12. Toward a Precision Medicine Approach

Precision medicine is an emerging approach to healthcare that aims to identify the appropriate intervention for a specific patient, based on the phenotypic characteristics of the individuals and their disease. There is increasing recognition that OSA is a highly heterogeneous disorder in terms of the pathways to the disease, the expression of the disease, and the response to treatment [114]. Individual differences include variability in genome, environment, and lifestyle of each person [63]. Precision sleep medicine represents a shift away from an outdated one-size-fits-all approach of treatment toward a tailored approach that acknowledges alternative methods of diagnosis and management [43]. Personalization of treatment is particularly important in OSA, since it is a chronic condition requiring long-term therapy. While it is important that the therapy effectively controls the disorder, the importance of patient acceptance for adherence and effectiveness must be emphasized. OA therapy is the leading alternative treatment to PAP and provides an important role for dentists in the management of OSA in collaboration with medical practitioners. The uncertainty around OA treatment efficacy has been a barrier to its prescription as first-line therapy. This has spurred a major research effort aimed at identifying predictors of treatment response, thereby promoting the ideal of personalized medicine in OA therapy.

## 13. Conclusions

Oral appliances are the main alternative to PAP for the treatment of OSA. There has been an expansion of the research evidence supporting the use of oral appliances in clinical practice, and key professional bodies now recommend oral appliances for use in patients with OSA who are intolerant to PAP therapy or prefer an alternative therapy. Patient preference is a key component of patient-centred care and has a pivotal role in treatment acceptance, usage, and health outcomes. OA therapy requires a multidisciplinary approach, involving a sleep physician and a dental practitioner with expertise in the management of sleep disorders, in order to achieve optimal outcomes for the patients. It is time for the sleep field to work collaboratively to address the barriers to the effective implementation of this evidence-based treatment into routine clinical practice.

## Figures and Tables

**Table 1:** OA therapy efficacy variation.

Treatment Response Definitions		All OSA	OSA Severity		
			Mild	Moderate	Severe
‘Complete response’	Treatment AHI < 5/h	36.5%	52.2%	38.3%	23.6%
‘Near-complete response’	Treatment AHI < 10/h + ≥ 50% AHI reduction	52.2%	52.2%	59.6%	42.1%
‘Partial response’	≥50% AHI reduction	63.8%	52.2%	64.8%	70.0%

These representative data of OA efficacy variation regard a sample of 425 OSA patients treated with a two-piece customised device set to the maximum comfortable protrusive limit [23]. These data represent an individual-level analysis of research participants in studies from a single research centre. No upper limits for apnoea–hypopnea index (AHI) or body mass index (BMI) were set as entry criteria for the studies. Proportions of responders are shown for three commonly used definitions based on changes in the AHI. OA: oral appliances; OSA: obstructive sleep apnoea.

**Table 2:** Comparison of published methods for the prediction of oral appliance treatment outcome.

Prediction Test	Range of Diagnostic Accuracy				Accuracy Classification	Applicability Concerns	Reference
	AUC	Sensitivity	Specificity	Accuracy			
Craniofacial (cephalometry)	0.73–0.86	0.96	0.72		Fair–Poor	Radiation, poor prediction	[30]
Clinical factors (age, BMI) and OSA severity	0.66			58%	Poor	Clinically applicable, but poor prediction	[23]
Obesity and Mallampati score		0.85	0.55			Easy to perform, no prospective studies	[31]
Clinical factors and craniofacial (cephalometry)	0.73	0.61–0.78	0.55–0.82	51%	Poor	Radiation, poor prediction	[32]
PAP optimal pressure	0.65–0.70	0.86–0.87	0.32–0.62		Poor	Requires available pressure value, clinically applicable but variation between studies	[33,34,35]
Spirometry	0.91	0.36–0.80	0.30–0.80	45–57%	Excellent–Poor	Excellent performance in derivation study, but poor on prospective validation	[36,37]
Drug-induced sleep endoscopy	0.82	0.49	0.78	58%	Good	Costly and not widely available	[19]
Awake nasendoscopy	0.74–0.87	0.65–0.88	0.68–0.80	80%	Good	Excellent only in a small study of Japanese patients	[38]
Site of pharyngeal collapse (multisensory catheter)		0.57–0.80	0.73–1.0		Good	Invasive, not clinically applicable	[39]
Remotely controlled mandibular protrusion sleep studies		0.60–0.86	0.89–0.92	88%	Good–Excellent	Excellent if based on ODI, good if based on AHI. Potentially poor if accounting for inconclusive tests	[28,40,41]
Pathophysiology (airway collapsibility and unstable ventilator control)	0.86–0.96	1.0	0.87	63%	Good	Small sample, no prospective validation, not clinically applicable	[42]

This table provides a summary of a range of prediction tools and their diagnostic accuracy. Accuracy classification is based on the receiver operating characteristic (ROC) area under the curve (AUC) as excellent (AUC 0.90–1.0), good (AUC 0.80–0.90), fair (AUC 0.70–0.80), poor (AUC 0.60–0.70). Table reproduced with permission from [43]. ODI: Oxygen Desaturation Index; PAP: positive airway pressure.
